# The diagnostic accuracy of serum and synovial inflammatory markers in chronic periprosthetic joint infection among anaemic patients

**DOI:** 10.1302/2046-3758.152.BJR-2025-0110.R1

**Published:** 2026-02-01

**Authors:** Abudousaimi Aimaiti, Wentao Guo, Boyong Xu, Wenbo Mu, Tuerhongjiang Wahafu, Chen Zou, Long Hua, Li Cao

**Affiliations:** 1 Department of Orthopaedics, First Affiliated Hospital of Xinjiang Medical University, Urumqi, China

**Keywords:** Diagnostic accuracy, Inflammatory markers, Periprosthetic joint infection, Anaemic patients, inflammatory markers, Serum, Periprosthetic joint infection (PJI), Anaemia, interleukin-6, CRP, biomarkers, Synovial fluid, fibrinogen, white blood cell

## Abstract

**Aims:**

Preoperative anaemia is common in patients undergoing revision total joint replacement (TJR), yet its effects on inflammatory markers for diagnosing chronic periprosthetic joint infection (PJI) are poorly understood. This study aimed to investigate how preoperative anaemia impacts inflammatory biomarkers, and to establish anaemia-adjusted diagnostic thresholds for PJI.

**Methods:**

This retrospective cohort study was conducted at a tertiary referral centre, evaluating 886 revision arthroplasty cases (396 PJI and 490 aseptic failures) between January 2008 and October 2023. Serum biomarkers (CRP, ESR, D-dimer, fibrinogen, fibrin degradation products (FDP), procalcitonin, and interleukin-6 (IL-6)) and synovial fluid markers (white blood cell count (SF-WBC) and polymorphonuclear percentage (SF-PMN)) were analyzed. The diagnostic performance of these markers was assessed using receiver operating characteristic (ROC) curve analysis, with patients stratified by anaemia status.

**Results:**

Preoperative anaemia was present in 55.1% (218/396) of patients with chronic PJI. In anaemic patients, serum biomarker levels were significantly higher than those of non-anaemic patients: CRP (28.35 vs 14.75 mg/l; p < 0.001), ESR (58 vs 40 mm/h; p < 0.001), D-dimer (615 vs 415 ng/ml; p < 0.001), and IL-6 (22.19 vs 10.74 pg/ml; p < 0.001). After adjusting diagnostic thresholds for anaemia, the area under the curve (AUC) for CRP improved from 0.838 to 0.927. Similar improvements were observed for ESR and IL-6. Fibrinogen and FDP demonstrated moderate diagnostic utility, while procalcitonin showed limited diagnostic value in both anaemic and non-anaemic patients.

**Conclusion:**

Preoperative anaemia is associated with statistically significant increases in most inflammatory biomarker levels and with higher diagnostic thresholds in chronic PJI. Anaemia-adjusted cut-off values for CRP, ESR, and IL-6 may enhance diagnostic accuracy for PJI in this patient population.

Cite this article: *Bone Joint Res* 2026;15(2):99–112.

## Article focus

- This study quantified preoperative anaemia’s effect on serum (CRP, ESR, IL-6, D-dimer) and synovial biomarkers (white blood cell count (SF-WBC), polymorphonuclear percentage (PMN%)) in chronic periprosthetic joint infection (PJI).
- Established anaemia-adjusted diagnostic thresholds (CRP ≥ 28.35 mg/l, ESR ≥ 58 mm/h) with improved accuracy (change in area under the curve (ΔAUC): CRP +0.089; interleukin-6 (IL-6) +0.107).
- First unified framework resolving anaemia-related diagnostic contradictions, affirming synovial marker robustness (SF-WBC AUC = 0.889).

## Key messages

- Preoperative anaemia elevated serum biomarkers (CRP 28.35 vs 14.75 mg/l; ESR 58 vs 40 mm/h), reducing accuracy of conventional thresholds (CRP AUC 0.838 vs 0.927).
- D-dimer (AUC 0.653) and fibrinogen (AUC 0.784) underperformed; synovial markers remained superior (SF-WBC AUC 0.889).
- Anaemia-adjusted CRP achieved highest accuracy (AUC 0.927), reinforcing its role as a first-line tool.

## Strengths and limitations

- This study included the largest PJI biomarker–anaemia cohort to date (n = 886 revisions, 496 PJIs) and applied International Consensus Meeting-aligned thresholds derived from receiver operating characteristic-based and stratified validation, providing guideline-directed cutoff recommendations.
- Limitations include the single-centre design without stratification of anaemia subtypes, persistent discordance between D-dimer and fibrinogen performance (AUC < 0.80), lack of assessment of novel synovial markers (CRP/α-defensin), and absence of stratification by anaemia severity and aetiology.

## Introduction

Periprosthetic joint infection (PJI) remains a devastating complication of total joint replacement (TJR), affecting 1% to 2% of primary procedures.^1,2^ The burden of PJI is expected to increase proportionally with the rising number of arthroplasty procedures performed in an ageing population.^3,4^

Diagnosing PJI remains challenging, with various diagnostic tools and biomarkers being explored, and early and accurate PJI diagnosis is critical to guiding timely intervention.^5^ Current first-line biomarkers for diagnosing PJI, including CRP and ESR, exhibit variable sensitivity (CRP: 42% to 94%; ESR: 74% to 94%) and may yield false-negative results in up to 35% of patients with chronic infections, immunocompromised conditions, or hepatitis.^6-9^ Recent studies have sought to enhance diagnostic accuracy by exploring alternative approaches, such as the albumin-to-globulin ratio and CRP-to-albumin ratio.^10^ However, these novel combinations have not yet demonstrated superior performance compared to conventional markers. Coagulation parameters, including fibrinogen, D-dimer, and fibrin degradation products (FDP), have been proposed as potential adjuncts for diagnosing PJI.^11,12^ However, the evidence regarding their utility remains inconsistent. While meta-analyses suggest that D-dimer may outperform conventional markers,^13^ other studies have reported limited diagnostic value for coagulation markers, including FDP and D-dimer, in PJI diagnosis.^14-16^ Synovial fluid analysis, including white blood cell count (SF-WBC) and polymorphonuclear percentage (SF-PMN), remains a cornerstone in the diagnosis of PJI. Recent studies have further explored the diagnostic potential of absolute synovial polymorphonuclear neutrophil (PMN) count, as well as synovial fluid neutrophil extracellular traps, highlighting their utility as novel biomarkers.^17,18^

Preoperative anaemia is prevalent among patients undergoing major orthopaedic procedures, with reported incidence rates of 15% to 52% in primary arthroplasty and 46.6% in revision procedures.^19-24^ This condition has been consistently associated with adverse postoperative outcomes and increased perioperative complications. Recent observational analyses have demonstrated that anaemia significantly affects the expression of inflammatory markers, particularly serum CRP and interleukin-6 (IL-6).^25,26^ However, research examining the influence of preoperative anaemia on inflammatory marker expression remains limited, and the absence of established diagnostic thresholds for anaemic patients impedes improvements in diagnostic accuracy within clinical practice. This study had two primary objectives: 1) to investigate the influence of preoperative anaemia on conventional inflammatory markers, including CRP, ESR, D-dimer, fibrinogen, FDP, procalcitonin, IL-6, SF-WB, and PMN%; and 2) to establish anaemia-adjusted diagnostic thresholds for these markers to improve diagnostic accuracy in anaemic patients undergoing revision arthroplasty.

## Methods

### Study population

After obtaining institutional review board approval, we conducted a retrospective cohort study of 886 patient records from a large tertiary institution. The study cohort included 396 patients diagnosed with chronic PJI and 490 patients without PJI, representing aseptic revision cases. The study investigated patients who underwent preoperative aspiration prior to revision total hip (THA) or knee arthroplasty (TKA) between January 2008 and October 2023. A PJI was defined using the 2018 International Consensus Meeting (ICM) criteria.^11^ Aseptic revisions were defined as cases that did not meet the PJI criteria and involved revision arthroplasty for non-infectious causes, such as loosening, wear, or instability.^27^ Patients with a megaprosthesis, native-joint septic arthritis, or who did not meet the PJI criteria were excluded. Patients with underlying inflammatory conditions, such as rheumatoid arthritis (RA) or systemic lupus erythematosus (SLE), were also excluded. Additionally, cases of acute haematogenous PJI, defined as acute symptoms lasting less than three weeks and occurring more than three months after the index surgery, were excluded.^28^ Anaemia was defined based on population-specific risk thresholds, with haemoglobin levels < 13.0 g/dl for men and < 11.5 g/dl for women.^29^

### Procedure and measures

Data obtained from all patients including age, sex, BMI, procedure type (THA vs TKA), synovial aspirate results, serum blood studies, and culture results were retrieved from the electronic medical record. On the morning of the first day after admission, serum biomarkers were collected and analyzed, including serum ESR, CRP, IL-6, fibrinogen, FDP, procalcitonin, and D-dimer. Subsequently, synovial fluid was obtained prior to revision surgery for the examination of synovial fluid white blood cell count (SF-WBC) and polymorphonuclear neutrophil percentage (PMN%), as well as for culture. Histological analysis of periprosthetic tissue for polymorphonuclear neutrophils (PMNs per high-power field, HPF) was also carried out.

### Statistical analysis

All statistical analyses were performed using SPSS version 27.0 (IBM, USA). A two-sided p-value < 0.05 was considered statistically significant. Continuous variables were assessed for normality and are presented as means and SDs or medians and IQRs, as appropriate, while categorical variables are expressed as frequencies and percentages. Continuous variables were compared using the Mann–Whitney U test for two-group comparisons and the Kruskal–Wallis H-test for comparisons among three BMI groups. Categorical variables were analyzed using the chi-squared test or Fisher’s exact test, as appropriate. Receiver operating characteristic (ROC) curve analysis was performed to evaluate diagnostic performance, with the area under the curve (AUC) and 95% CIs calculated. Optimal cut-off values were determined using the Youden index. Sensitivity, specificity, positive predictive value (PPV), and negative predictive value (NPV) with 95% CIs were calculated. ROC curves and scatter plots were generated using GraphPad Prism software (version 10.41; GraphPad Software, USA).

## Results

**Fig. 1:**
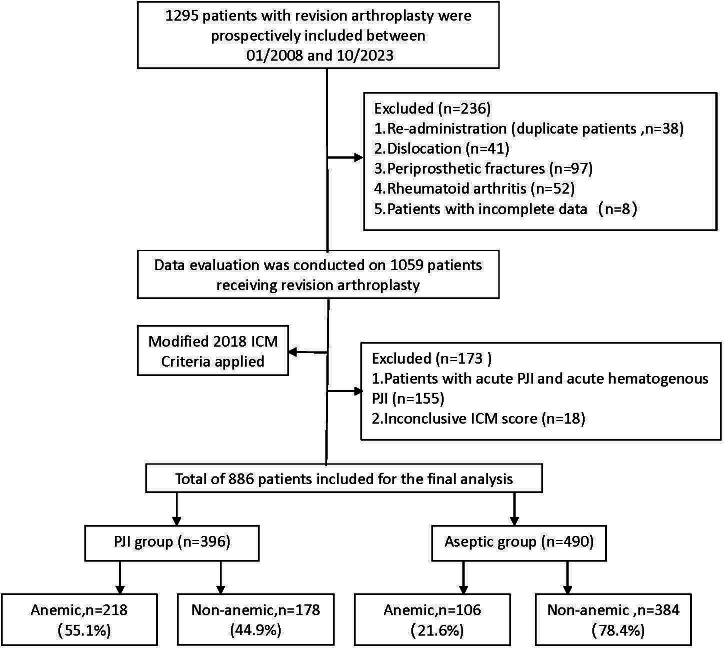
Flowchart of patient selection showing the stratification of 1,295 revision arthroplasty cases into periprosthetic joint infection (PJI) (n = 396) and aseptic (n = 490) groups, with further subdivision by anaemia status. ICM, International Consensus Meeting.

Preoperative anaemia was present in 55.1% (218/396) of patients with chronic PJI. The anaemic group demonstrated significantly lower BMI (median 21 kg/m² (IQR 20 to 29) vs 28 kg/m² (IQR 22 to 29); p < 0.001, Mann-Whitney U test) and higher rates of previous blood transfusion (24.7% vs 16.5%; p = 0.043, chi-squared test) compared with the non-anaemic group. Age, sex distribution, joint type, comorbidities, and other medical history factors were similar between groups (all p > 0.05) (Table I, Figure 1).

**Table I.** Comparison of demographic and clinical characteristics in patients with periprosthetic joint infection with and without anaemia.

Variable	Anaemic PJI (n = 218)	Non-anaemic PJI (n = 178)	χ² or Z	p-value
Sex (female/male), n	115/103	93/85	χ² = 0.368	0.602*
Median age, yrs (IQR)	67 (58 to 74)	65 (54 to 71)	Z = −1.810	0.070†
Joint (knee/hip), n	187/150	129/116	χ² = 0.460	0.498*
Median BMI, kg/m^2^ (IQR)	21 (20 to 29)	28 (22 to 29)	Z = -10.433	< 0.001†
Sinus tract, n (%)	87 (39.91)	63 (35.39)	χ² = 0.849	0.357*
Comorbidities, n (%)*				
Hypertension	85 (38.9)	56 (31.4)	χ² = 2.423	0.120
Diabetes	41 (18.8)	25 (14.0)	χ² = 1.600	0.206
Coronary heart disease	20 (9.1)	15 (8.4)	χ² = 0.068	0.794
Tuberculosis	12 (5.5)	12 (6.7)	χ² = 0.263	0.608
Cerebral infarction	7 (3.2)	10 (5.6)	χ² = 1.382	0.240
Tumour	14 (6.4)	6 (3.3)	χ² = 1.902	0.168
Past medical history, n (%)*				
Debridement	34 (15.5)	20 (11.2)	χ² = 1.582	0.208
Aseptic revision	10 (4.5)	6 (3.3)	χ² = 0.374	0.541
Blood transfusion	36 (16.5)	44 (24.7)	χ² = 4.092	0.043
Drug allergy	25 (11.4)	22 (12.3)	χ² = 2.423	0.120
Smoking	18 (8.2)	24 (13.4)	χ² = 0.001	0.978
Alcoholism	8 (3.6)	14 (7.8)	χ² = 3.287	0.070

* Chi-squared test.

† Mann-Whitney U test.

PJI, periprosthetic joint infection.

**Fig. 2:**
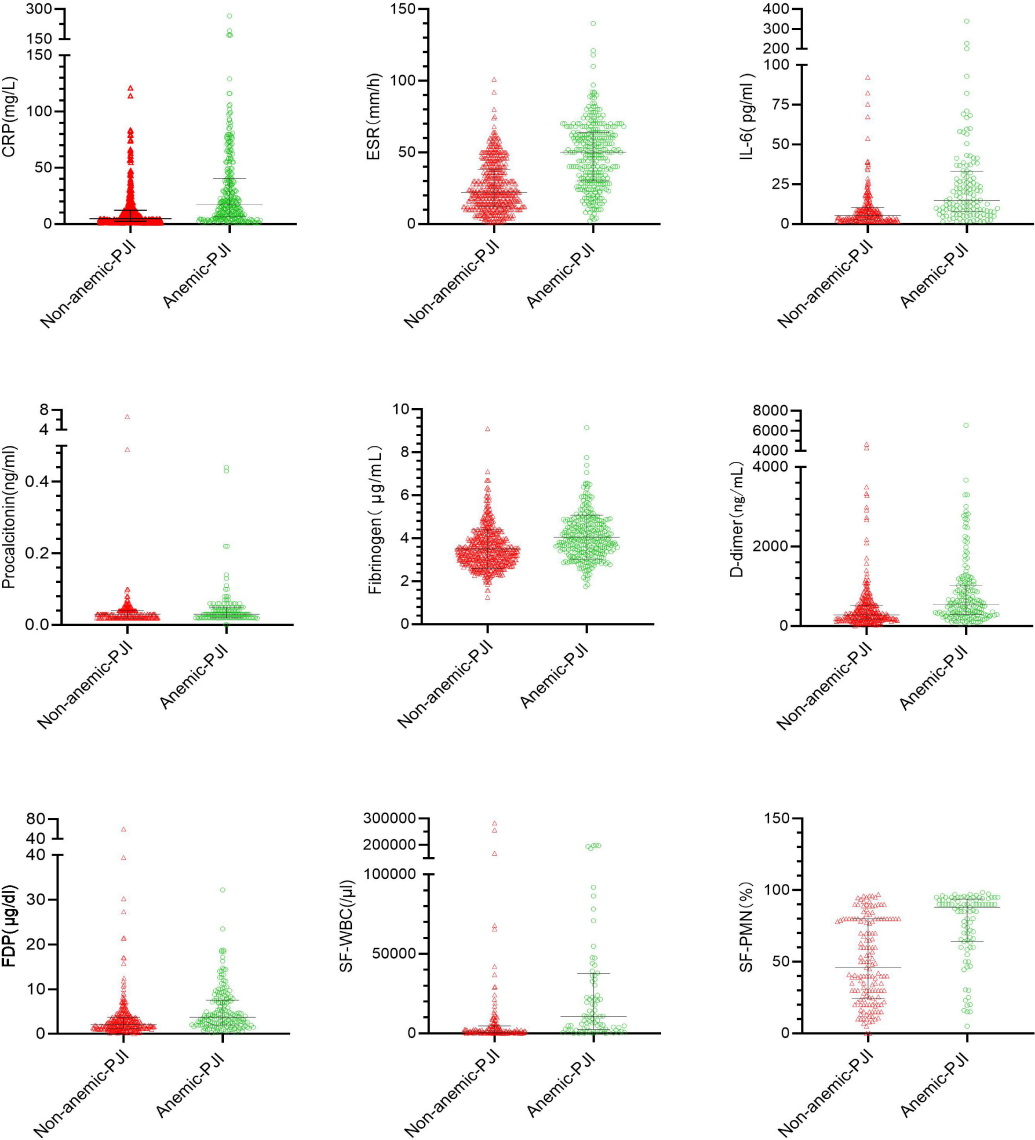
Scatter plots comparing serum inflammatory markers (CRP, ESR, interleukin-6 (IL-6), procalcitonin, fibrinogen, D-dimer, fibrin degradation products (FDP))) and synovial fluid parameters (SF-WBC, SF-PMN%) between anaemic (green) and non-anaemic (red) periprosthetic joint infection (PJI) patients. Horizontal lines represent median values. PMN, polymorphonuclear neutrophil percentage; SF, synovial fluid; WBC, white blood cell count.

Serum and synovial inflammatory markers were compared between patients with (n = 218) and without anaemia (n = 178) (Table II, Figure 2). Median CRP levels were significantly higher in the anaemic PJI group (28.35 mg/l (IQR 15.05 to 58.25)) than in the non-anaemic PJI group (14.75 mg/l (IQR 7.767 to 28.62)), with a statistically significant difference (p < 0.001, Mann-Whitney U test). Similarly, median ESR was elevated in the anaemic PJI group (58 mm/h (IQR 46 to 68)) compared with the non-anaemic group (40 mm/h (IQR 24 to 50)), demonstrating a highly significant difference (p < 0.001, Mann-Whitney U test). Median IL-6 levels were significantly higher in patients with anaemia (22.2 pg/ml (IQR 11.1 to 38.8)) compared with those without anaemia (10.7 pg/ml (IQR 7.5 to 19.0); p < 0.001, Mann-Whitney U test). Similarly, median D-dimer levels were elevated in the anaemic group (615 ng/ml (IQR 322 to 1,157)) compared with the non-anaemic group (415 ng/ml (IQR 206 to 737); p < 0.001, Mann-Whitney U test). Mean fibrinogen levels were slightly higher in the anaemic PJI group (4.33 g/l (SD 0.95)) than in the non-anaemic group (4.00 g/l (SD 1.06)), with no significant difference (t = 0.81, p = 0.369, independent-samples *t*-test). Median procalcitonin levels were similar in both groups (0.35 µg/l (IQR 0.200 to 0.500); p = 0.272, Mann-Whitney U test). Median FDP levels were significantly higher in the anaemic PJI group (4.21 µg/ml (IQR 2.30 to 8.70)) than in the non-anaemic group (2.95 µg/ml (1.68 to 5.27); p < 0.001). Median SF-WBC was elevated in the anaemic group (14,589 × 10⁹/l (IQR 4,850 to 44,160)) compared with the non-anaemic group (4,200 × 10⁹/l (IQR 1,195 to 10,810); p < 0.001). Median SF-PMN% was also higher in the anaemic group (90% (IQR 74 to 94)) than in the non-anaemic group (80% (IQR 50 to 89); p < 0.001).

**Table II.** Comparison of serum and synovial inflammatory markers in periprosthetic joint infection between anaemic and non-anaemic patients.

Variable	Anaemic PJI (n = 218)	Non-anaemic PJI (n = 178)	t or Z	p-value
Median CRP, mg/l (IQR)	28.35 (15.05 to 58.25)	14.75 (7.767 to 28.62)	Z = −6.333	< 0.001*
Median ESR, mm/h (IQR)	58 (46 to 68)	40 (24 to 50)	Z = −9.368	< 0.001*
Median D-dimer, ng/ml (IQR)	615 (322 to 1,157)	415 (206 to 737)	Z = −3.361	< 0.001*
Mean fibrinogen, g/l (SD)	4.33 (0.946)	4.00 (1.061)	t = 0.808	0.369†
Median IL-6, pg/ml (IQR)	22.19 (11.08 to 38.79)	10.74 (7.53 to 19.03)	Z = −4.623	< 0.001*
Median FDP, μg/ml (IQR)	4.21 (2.30 to 8.70)	2.95 (1.68 to 5.27)	Z = −3.190	< 0.001*
Median procalcitonin, μg/l (IQR)	0.35 (0.20 to 0.06)	0.35 (0.02 to 0.05)	Z = −1.099	0.272*
Median SF-WBC, cells/µl (IQR)	14,589 (4,850 to 44,160)	4200 (1,195 to 10,810)	Z = −4.119	< 0.001*
Median SF-PMN (IQR)	90 (74 to 94)	80 (50 to 89)	Z = −3.738	< 0.001*

* Mann-Whitney U test.

† Independent-samples *t*-test.

FDP, fibrin degradation products; IL-6, interleukin-6; PJI, periprosthetic joint infection; PMN, polymorphonuclear neutrophil percentage; SF, synovial fluid; WBC, white blood cell count.

**Fig. 3:**
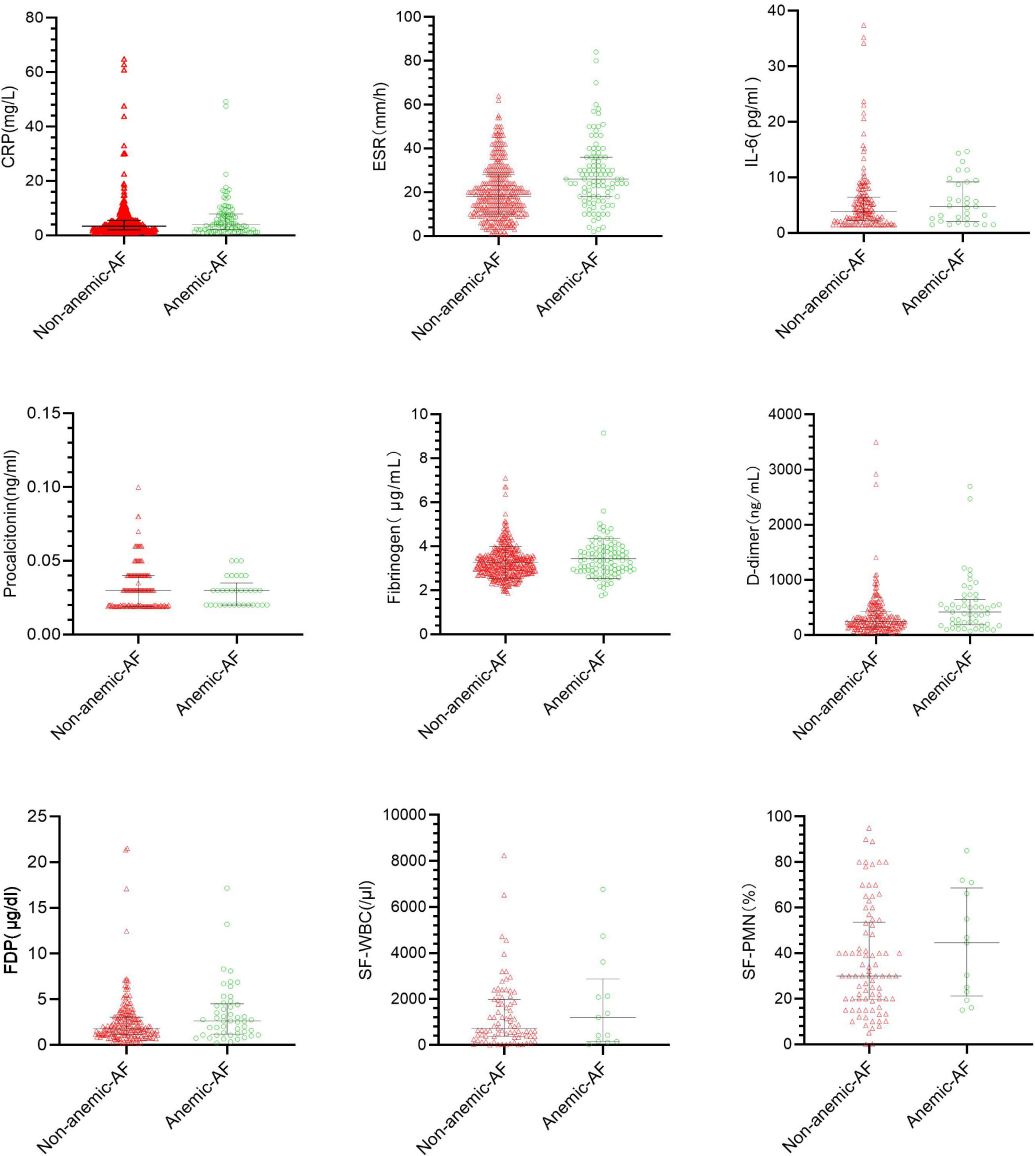
Scatter plots comparing serum inflammatory markers (CRP, ESR, interleukin-6 (IL-6), procalcitonin, fibrinogen, D-dimer, fibrin degradation products (FDP)) and synovial fluid parameters (SF-WBC, SF-PMN%) between anaemic (green) and non-anaemic (red) aseptic failure (AF) patients. Horizontal lines represent median values. PMN, polymorphonuclear neutrophil percentage; SF, synovial fluid; WBC, white blood cell count.

Significant differences in serum inflammatory markers were observed between the anaemic (n = 106) and non-anaemic (n = 384) aseptic failure cohorts (Table III, Figure 3). The anaemic group demonstrated markedly higher ESR (median 28 mm/h(IQR 13 to 36) vs mean 22.9 mm/h (SD 1.62); p < 0.001) and elevated median D-dimer levels (416 ng/ml (IQR 188 to 644) vs 244 ng/ml (IQR 141 to 425); p = 0.002, Mann-Whitney U test). Median fibrinogen levels were modestly increased in anaemic patients (3.36 g/l (IQR 2.87 to 3.86) vs 3.19 g/l (IQR 2.80 to 3.59); p = 0.049), and median FDP levels were significantly higher in the anaemic group (2.63 µg/ml (IQR 1.17 to 4.50) vs 1.78 µg/ml (IQR 1.12 to 3.05); p = 0.020, Mann–Whitney U test).

**Table III.** Comparison of serum and synovial inflammatory markers in aseptic failure between anaemic and non-anaemic patients.

Variable	Anaemic AF (n = 106)	Non-anaemic AF (n = 384)	Z or T	p-value
CRP (mg/l)	2.85 (1.36 to 6.75)	5.179 (SD 0.808)	Z = −1.530	0.126*
ESR (mm/h)	28 (13 to 35)	22.90 (SD 1.62)	Z = −5.176	< 0.001*
D-dimer (ng/ml)	416 (188 to 644)	244 (141 to 425)	Z = −3.038	0.002*
Fibrinogen (g/l)	3.36 (2.87 to 3.86)	3.19 (2.80 to 3.59)	Z = −1.970	0.049*
IL-6 (pg/ml)	4.70 (2.10 to 9.19)	5.60 (SD 0.47)	Z = −0.977	0.329*
FDP (μg/ml)	2.63 (1.17 to 4.50)	1.78 (1.12 to 3.05)	Z = −2.326	0.020*
Median procalcitonin, μg/l (IQR)	0.03 (0.02 to 0.03)	0.03 (0.01 to 0.04)	Z = −0.607	0.544*
Mean SF-WBC, cells/µl (SD)	1786.15 (SD 579.98)	838.50 (380.00 to 2262.50)	Z = −0.967	0.967*
Mean SF-PMN (SD)	43.81 (SD 24.21)	36.57 (SD 23.71)	T = −1.022	0.265†

Normality was assessed using the Shapiro–Wilk test. CRP, ESR, and IL-6 were normally distributed in the non-anaemic group (p > 0.05) and are therefore presented as mean (SD), whereas they were non-normally distributed in the anaemic group and are presented as median (IQR). SF-WBC was normally distributed in the anaemic group and is presented as mean (SD). All other variables were non-normally distributed in both groups and are expressed as median (IQR), except neutrophil percentage, which is presented as mean (SD). Accordingly, between-group comparisons were performed using the independent-samples *t*-test for neutrophil percentage and the Mann–Whitney U test for all other variables.

* Mann-Whitney U test.

† Independent-samples *t*-test.

AF, aseptic failure; FDP, fibrin degradation products; IL-6, interleukin-6; PMN, polymorphonuclear neutrophil percentage; SF, synovial fluid; WBC, white blood cell count.

**Fig. 4:**
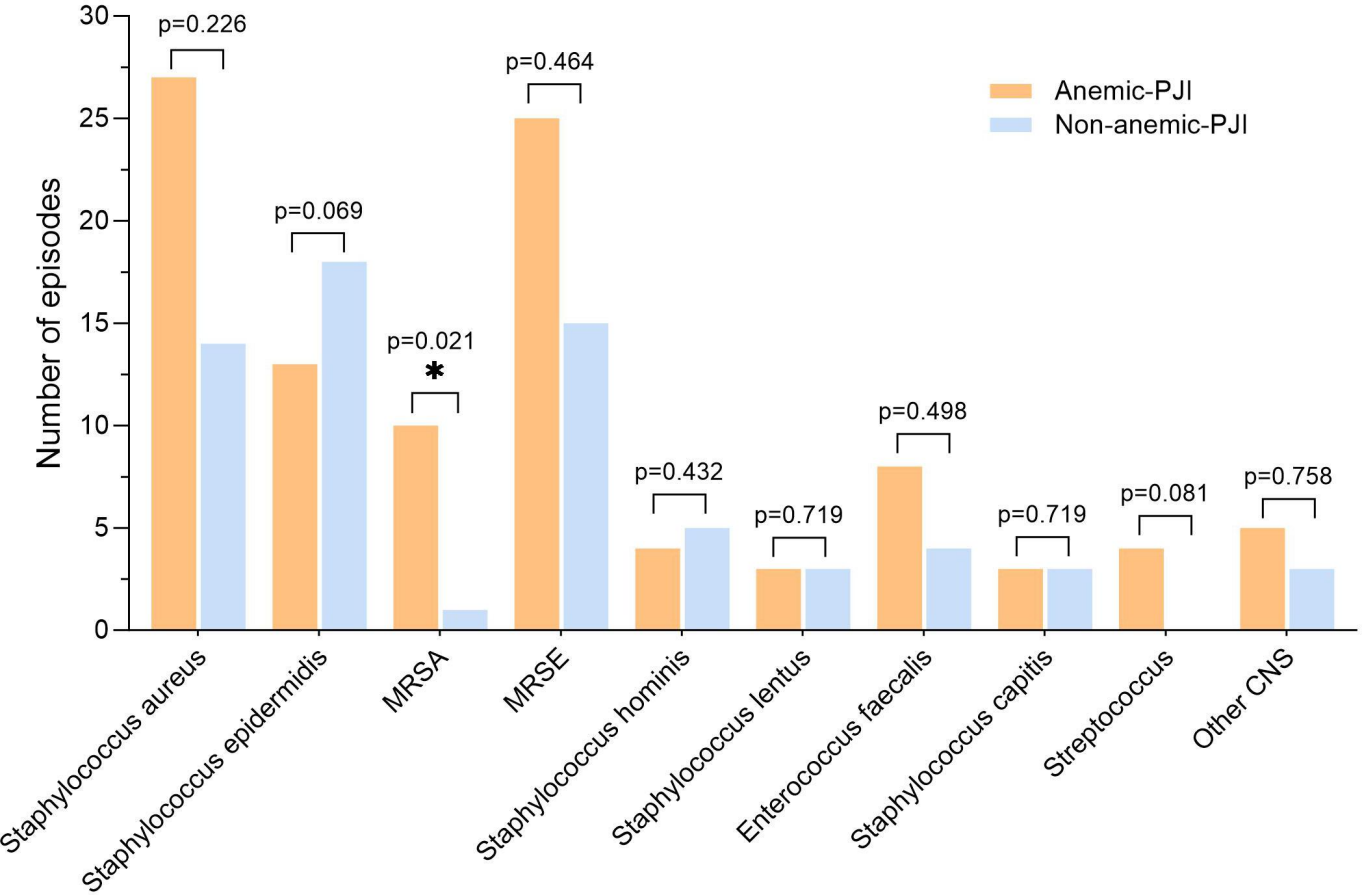
Comparison of Gram-positive bacterial species distribution between anaemic (orange bars) and non-anaemic (blue bars) periprosthetic joint infection (PJI) patients. *Significant difference for methicillin-resistant *Staphylococcus aureus* (MRSA) (p = 0.021, chi-squared test). CNS, coagulase-negative staphylococci; MRSE, methicillin-resistant *Staphylococcus epidermidis*.

**Fig. 5:**
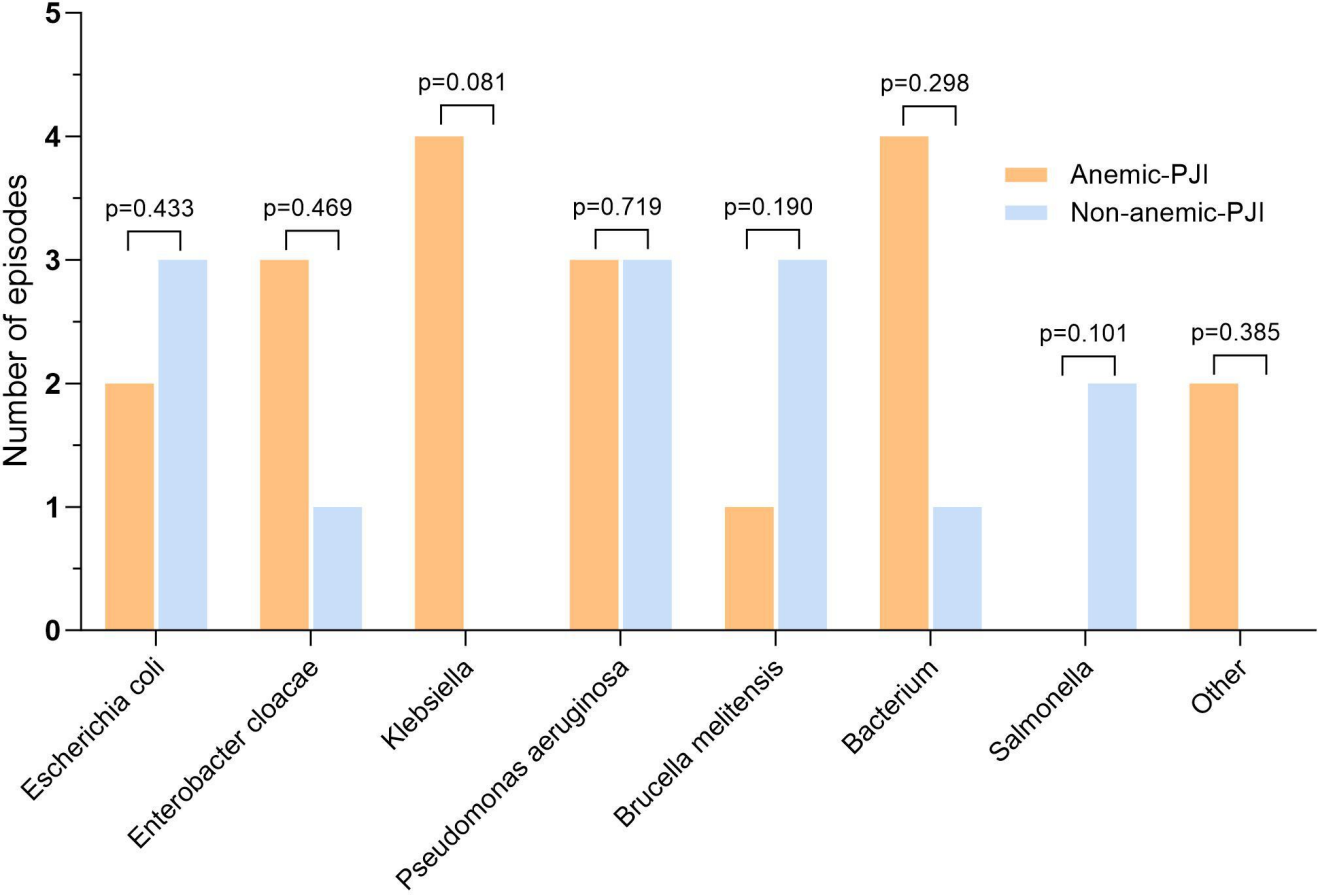
Distribution of Gram-negative bacterial species between anaemic (orange bars) and non-anaemic (blue bars) periprosthetic joint infection (PJI) patients. No significant differences were observed between groups (all p > 0.05, chi-squared test).

**Fig. 6:**
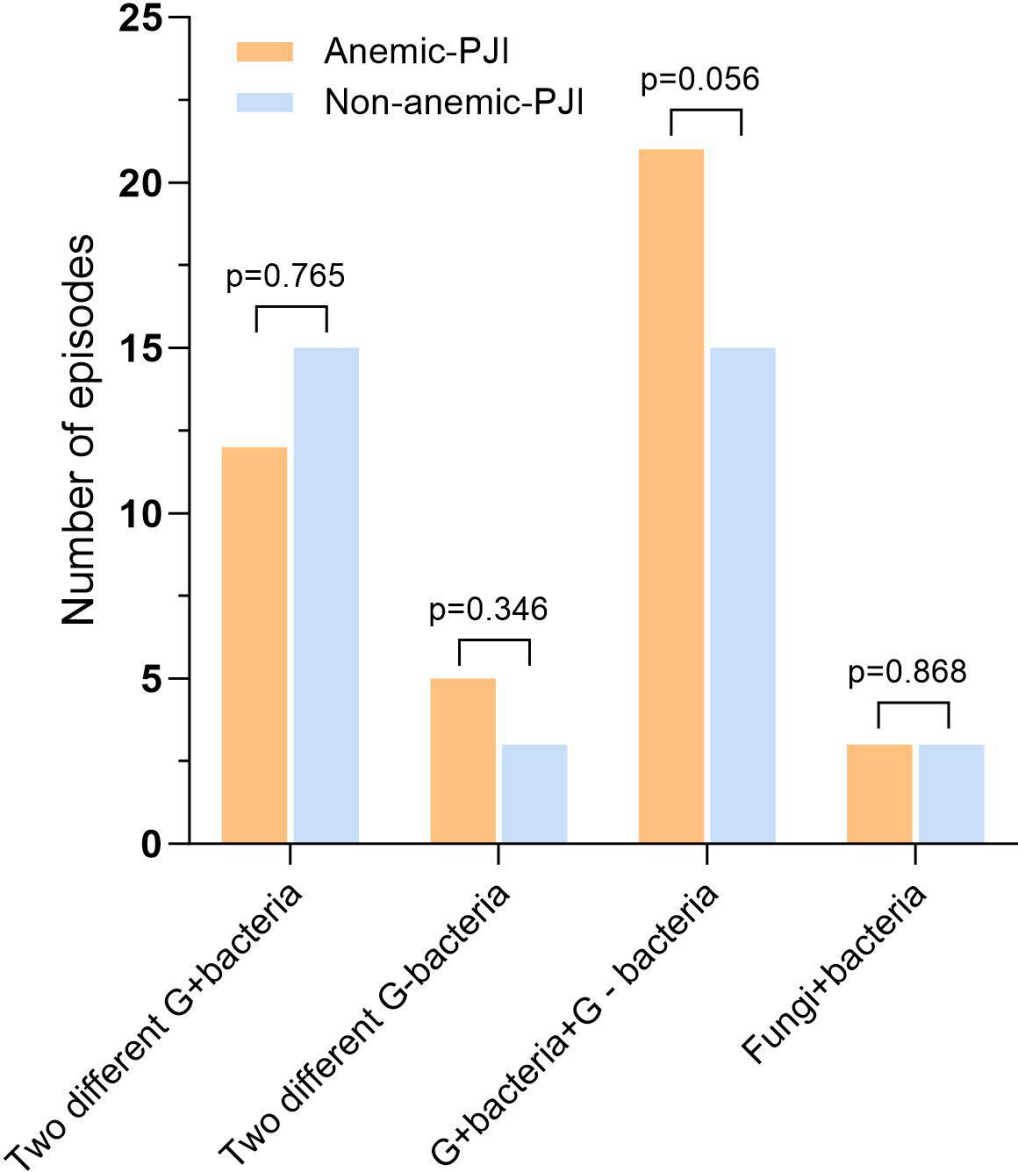
Patterns of polymicrobial infections between anaemic (orange bars) and non-anaemic (blue bars) periprosthetic joint infection (PJI) patients, including combinations of Gram-positive (G+), Gram-negative (G-), and fungal organisms. No significant differences were observed between groups (all p > 0.05, chi-squared test).

No significant differences were found in CRP (median 2.85 mg/l vs mean 5.18 mg/l; p = 0.126), IL-6 (median 4.70 pg/ml vs mean 5.60 pg/ml; p = 0.329), procalcitonin (median 0.03 µg/l vs 0.03 µg/l; p = 0.544), SF-WBC (mean 1,786.15 × 10⁹/l vs median 838.50 × 10⁹/l; p = 0.967), or SF-PMN% (mean 43.81% vs 36.57%; p = 0.265). Microbiological analysis (Table IV, Figures 4 to 6) demonstrated that Gram-positive organisms were the predominant pathogens in both anaemic and non-anaemic PJI groups (62.6% (n = 102) vs 54.1% (n = 66); p = 0.150). Notably, methicillin-resistant *Staphylococcus aureus* (MRSA) infections were significantly more frequent in anaemic patients compared with non-anaemic patients (6.1% (n = 10) vs 0.8% (n = 1); p = 0.021). Conversely, polymicrobial infections were significantly more prevalent in the non-anaemic group (33.6% (n = 41) vs 22.1% (n = 36); p = 0.030).

**Table IV.** Microbiological profile of periprosthetic joint infections in anaemic and non-anaemic patients.

Episodes	Anaemic PJI patients, n (%)	Non-anaemic PJI patients, n (%)	p-value*
Gram-positive bacteria	102 (62.6)	66 (54.1)	0.150
*Staphylococcus aureus*	27 (16.6)	14 (11.5)	0.226
*Staphylococcus epidermidis*	13 (7.9)	18 (14.4)	0.069
MRSA	10 (6.1)	1 (0.8)	0.021
MRSE	25 (15.3)	15 (12.3)	0.464
*Staphylococcus hominis*	4 (2.5)	5 (4.1)	0.432
*Staphylococcus lentus*	3 (1.8)	3 (2.5)	0.719
*Enterococcus faecalis*	8 (4.9)	4 (3.3)	0.498
*Staphylococcus capitis*	3 (1.8)	3 (2.5)	0.719
*Streptococcus*	4 (2.5)	0 (0.0)	0.081
Other CNS	5 (3.1)	3 (2.5)	0.758
Gram-negative bacteria	19 (11.7)	13 (10.7)	0.791
*Escherichia coli*	2 (1.2)	3 (2.5)	0.433
*Enterobacter cloacae*	3 (1.8)	1 (0.8)	0.469
*Klebsiella*	4 (2.5)	0 (0.0)	0.081
*Pseudomonas aeruginosa*	3 (1.8)	3 (2.5)	0.719
*Brucella melitensis*	1 (0.6)	3 (2.5)	0.190
Bacterium	4 (2.5)	1 (0.8)	0.298
*Salmonella*	0 (0.0)	2 (1.6)	0.101
Other	2 (1.2)	0 (0.0)	0.385
Polymicrobial	36 (22.1)	41 (33.6)	0.030
Two different G+ bacteria	12 (7.36)	15 (12.3)	0.765
Two different G- bacteria	5 (3.1)	3 (2.5)	0.346
G+ and G- bacteria	21 (12.8)	15 (12.3)	0.056
Fungi+ bacteria	3 (1.8)	3 (2.5)	0.868
Total	163	122	

* Chi-squared test.

CNS, coagulase-negative staphylococci; G+, Gram-positive; G-, Gram-negative; MRSA, methicillin-resistant *Staphylococcus aureus*; MRSE, methicillin-resistant *Staphylococcus epidermidis*; PJI, periprosthetic joint infection.

**Fig. 7:**
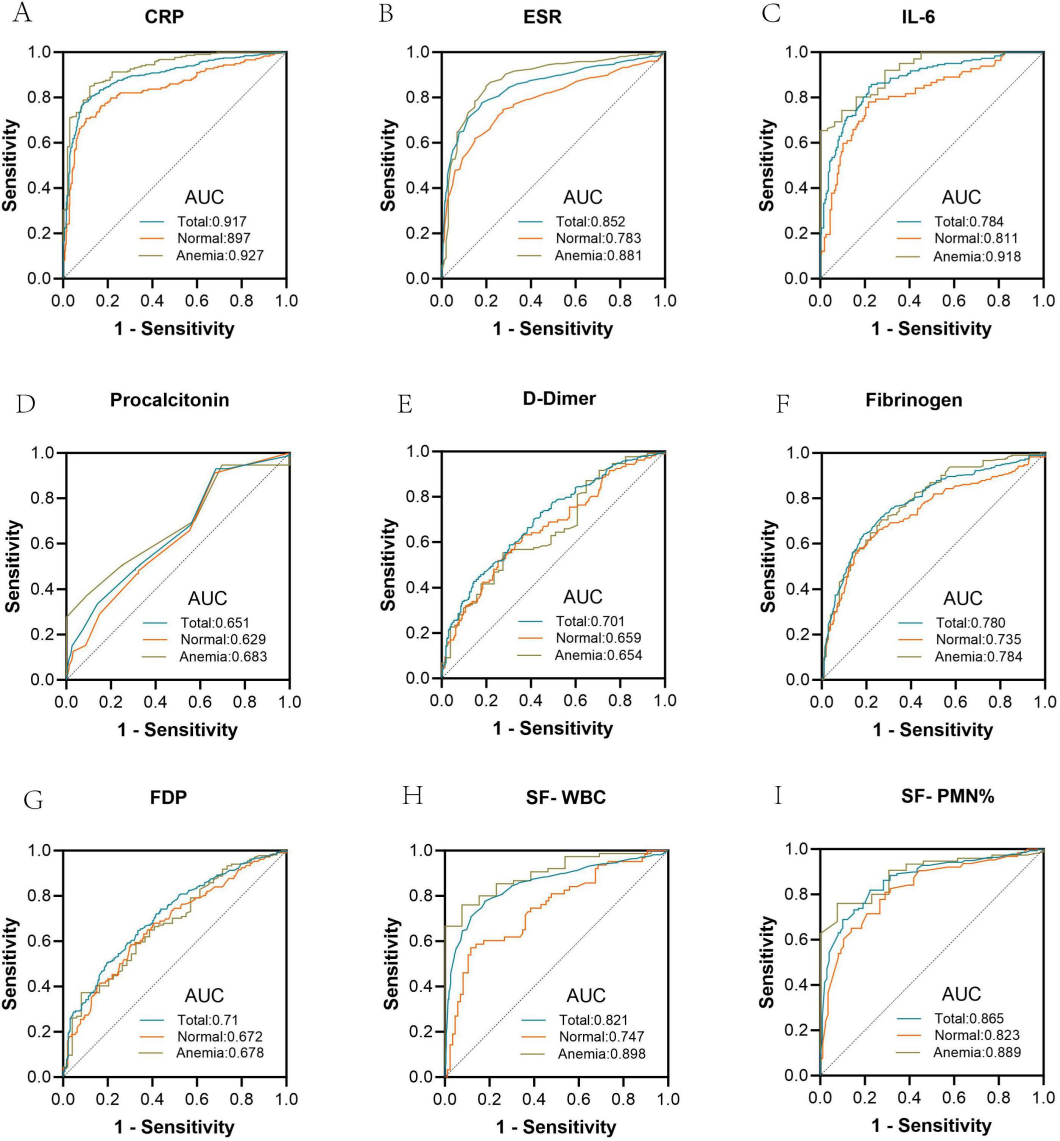
Receiver operating characteristic (ROC) curves comparing diagnostic performance of inflammatory markers in chronic periprosthetic joint infection (PJI) between total population (blue), non-anaemic (orange), and anaemic (green) patients. Area under the curve (AUC) values are shown for serum biomarkers (CRP, ESR, interleukin-6 (IL-6), procalcitonin, D-dimer, fibrinogen, fibrin degradation products (FDP)) and synovial fluid parameters (white blood cell count (SF-WBC), polymorphonuclear neutrophil percentage (SF-PMN%)).

ROC analysis (Table V, Figure 7) showed that CRP provided excellent discrimination for PJI in the overall cohort (AUC 0.893), with an optimal cut-off of 11.05 mg/l yielding a sensitivity of 0.765 and a specificity of 0.920. Diagnostic accuracy was higher in anaemic than in non-anaemic patients (AUC 0.927 vs 0.838). ESR also demonstrated good overall accuracy (AUC 0.852) at a cut-off of 32.5 mm/h (sensitivity 0.775; specificity 0.828), again showing higher discrimination in anaemic patients (AUC 0.881 vs 0.782). IL-6 achieved strong overall performance (AUC 0.873) with a cut-off of 7.13 µg/l (sensitivity 0.857; specificity 0.771), and showed improved discrimination in anaemic compared with non-anaemic patients (AUC 0.918 vs 0.811), with a higher optimal threshold in the anaemic subgroup (14.85 µg/l). In contrast, procalcitonin demonstrated limited overall discrimination (AUC 0.651) despite high sensitivity at its optimal cut-off of 0.02 µg/l (sensitivity 0.929; specificity 0.550), with marked differences in the sensitivity–specificity trade-off between subgroups. D-dimer showed moderate diagnostic accuracy (AUC 0.700) at a cut-off of 319 ng/ml (sensitivity 0.701; specificity 0.587), and did not demonstrate improved performance in the anaemic subgroup (AUC 0.653 vs 0.659). Fibrinogen (AUC 0.780; cut-off 3.805 g/l; sensitivity 0.638; specificity 0.811) and FDP (AUC 0.710; cut-off 2.66 µg/ml; sensitivity 0.645; specificity 0.665) demonstrated modest diagnostic discrimination. Among synovial biomarkers, SF-WBC (AUC 0.821; cut-off 3633 ×10⁹/l; sensitivity 0.688; specificity 0.878) and SF-PMN% (AUC 0.865; cut-off 57.85%; sensitivity 0.818; specificity 0.777) showed strong overall diagnostic performance, with the highest AUCs observed in anaemic PJI patients.

**Table V.** Diagnostic accuracy of each serological and synovial marker using receiver operating characteristic curve analyses.

Parameters	Total	Non-anaemic PJI	Anaemic PJI
**CRP**			
AUC (95% CI)	0.893 (0.870 to 0.915)	0.838 (0.798 to 0.878)	0.927 (0.897 to 0.956)
Cutoff, mg/l	11.05*	9.53*	11.05*
Sensitivity (95% CI)	0.765 (0.719 to 0.805)	0.707 (0.634 to 0.772)	0.848 (0.792 to 0.892)
Specificity (95% CI)	0.920 (0.891 to 0.942)	0.901 (0.865 to 0.928)	0.886 (0.806 to 0.937)
PPV, % (95% CI)	0.885 (0.846 to 0.916)	0.768 (0.694 to 0.828)	0.939 (0.893 to 0.966)
NPV, % (95% CI)	0.829 (0.794 to 0.859)	0.869 (0.831 to 0.900)	0.740 (0.653 to 0.812)
**ESR**			
AUC (95% CI)	0.852 (0.825 to 0.879)	0.782 (0.737 to 0.827)	0.881 (0.839 to 0.922)
Cutoff, mm/h	32.5*	24.5*	38.5*
Sensitivity (95% CI)	0.775 (0.730 to 0.814)	0.747 (0.675 to 0.807)	0.848 (0.792 to 0.892)
Specificity (95% CI)	0.828 (0.791 to 0.860)	0.734 (0.686 to 0.777)	0.811 (0.721 to 0.878)
PPV, % (95% CI)	0.785 (0.740 to 0.824)	0.565 (0.499 to 0.629)	0.902 (0.851 to 0.937)
NPV, % (95% CI)	0.820 (0.782 to 0.852)	0.862 (0.819 to 0.896)	0.722 (0.631 to 0.798)
**IL-6**			
AUC (95% CI)	0.873 (0.838 to 0.909)	0.811 (0.751 to 0.871)	0.918 (0.869 to 0.967)
Cutoff, μg/l	7.13*	7.13*	14.85*
Sensitivity (95% CI)	0.857 (0.796 to 0.903)	0.780 (0.672 to 0.861)	0.653 (0.551 to 0.743)
Specificity (95% CI)	0.771 (0.703 to 0.827)	0.783 (0.709 to 0.843)	1.000 (0.862 to 1.000)
PPV, % (95% CI)	0.785 (0.720 to 0.838)	0.653 (0.549 to 0.744)	1.000 (0.931 to 1.000)
NPV, % (95% CI)	0.847 (0.783 to 0.896)	0.872 (0.803 to 0.921)	0.469 (0.347 to 0.595)
**Procalcitonin**			
AUC (95% CI)	0.651 (0.595 to 0.706)	0.629 (0.556 to 0.702)	0.682 (0.585 to 0.780)
Cutoff, μg/l	0.02*	0.0195*	0.045*
Sensitivity (95% CI)	0.929 (0.865 to 0.965)	0.911 (0.820 to 0.960)	0.372 (0.276 to 0.478)
Specificity (95% CI)	0.550 (0.484 to 0.613)	0.335 (0.264 to 0.414)	0.909 (0.745 to 0.976)
PPV, % (95% CI)	0.522 (0.454 to 0.588)	0.402 (0.330 to 0.478)	0.921 (0.775 to 0.979)
NPV, % (95% CI)	0.936 (0.878 to 0.968)	0.885 (0.771 to 0.948)	0.337 (0.242 to 0.445)
**D-dimer**			
AUC (95% CI)	0.700 (0.655 to 0.746)	0.659 (0.594 to 0.724)	0.653 (0.567 to 0.741)
Cutoff, ng/ml	319*	319*	559.5*
Sensitivity (95% CI)	0.701 (0.638 to 0.758)	0.632 (0.532 to 0.722)	0.553 (0.464 to 0.638)
Specificity (95% CI)	0.587 (0.524 to 0.647)	0.635 (0.565 to 0.701)	0.725 (0.580 to 0.836)
PPV, % (95% CI)	0.611 (0.551 to 0.669)	0.471 (0.388 to 0.557)	0.839 (0.741 to 0.906)
NPV, % (95% CI)	0.680 (0.613 to 0.740)	0.771 (0.698 to 0.829)	0.385 (0.289 to 0.491)
**Fibrinogen**			
AUC (95% CI)	0.780 (0.748 to 0.811)	0.735 (0.687 to 0.783)	0.784 (0.730 to 0.838)
Cutoff, g/l	3.805*	3.805*	3.785*
Sensitivity (95% CI)	0.638 (0.588 to 0.686)	0.581 (0.503 to 0.655)	0.699 (0.632 to 0.759)
Specificity (95% CI)	0.811 (0.772 to 0.844)	0.829 (0.786 to 0.865)	0.732 (0.633 to 0.813)
PPV, % (95% CI)	0.729 (0.678 to 0.775)	0.606 (0.526 to 0.680)	0.846 (0.782 to 0.894)
NPV, % (95% CI)	0.737 (0.697 to 0.774)	0.814 (0.771 to 0.851)	0.536 (0.449 to 0.620)
**FDP**			
AUC (95% CI)	0.710 (0.665 to 0.755)	0.672 (0.608 to 0.736)	0.677 (0.592 to 0.763)
Cutoff, μg/ml	2.66*	2.615*	6.925*
Sensitivity (95% CI)	0.645 (0.581 to 0.705)	0.584 (0.485 to 0.678)	0.373 (0.292 to 0.461)
Specificity (95% CI)	0.665 (0.603 to 0.722)	0.697 (0.628 to 0.758)	0.918 (0.795 to 0.973)
PPV, % (95% CI)	0.645 (0.581 to 0.705)	0.500 (0.409 to 0.590)	0.925 (0.812 to 0.976)
NPV, % (95% CI)	0.665 (0.603 to 0.722)	0.764 (0.696 to 0.822)	0.348 (0.268 to 0.438)
**SF-WBC**			
AUC (95% CI)	0.821 (0.767 to 0.875)	0.747 (0.666 to 0.828)	0.898 (0.827 to 0.970)
Cutoff, ×10^9^/l	3,633*	3,237*	4,795*
Sensitivity (95% CI)	0.688 (0.603 to 0.762)	0.571 (0.440 to 0.693)	0.760 (0.645 to 0.847)
Specificity (95% CI)	0.878 (0.794 to 0.933)	0.883 (0.792 to 0.939)	0.923 (0.620 to 0.995)
PPV, % (95% CI)	0.887 (0.808 to 0.938)	0.782 (0.632 to 0.885)	0.982 (0.895 to 0.999)
NPV, % (95% CI)	0.669 (0.580 to 0.747)	0.737 (0.640 to 0.817)	0.400 (0.232 to 0.592)
**SF-PMN**			
AUC (95% CI)	0.865 (0.818 to 0.911)	0.823 (0.754 to 0.892)	0.889 (0.813 to 0.964)
Cutoff, %	57.85*	43.5*	73*
Sensitivity (95% CI)	0.818 (0.742 to 0.877)	0.809 (0.687 to 0.893)	0.760 (0.645 to 0.847)
Specificity (95% CI)	0.777 (0.680 to 0.852)	0.709 (0.599 to 0.799)	0.923 (0.620 to 0.995)
PPV, % (95% CI)	0.837 (0.761 to 0.892)	0.671 (0.552 to 0.771)	0.982 (0.895 to 0.999)
NPV, % (95% CI)	0.754 (0.657 to 0.832)	0.835 (0.726 to 0.908)	0.400 (0.232 to 0.592)

* Calculated using the Youden index.

AUC, area under the curve; NPV, negative predictive value; PJI, periprosthetic joint infection; PMN, polymorphonuclear neutrophil percentage; PPV, positive predictive value; SF, synovial fluid; WBC, white blood cell count.

## Discussion

Management of PJI often involves complex surgical strategies, including the debridement, antibiotics, and implant retention (DAIR) protocol, as well as single- and two-stage revision arthroplasty; their efficacy and outcomes have been extensively studied.^30-33^ Preoperative anaemia, observed in nearly half of patients undergoing TJR, is well documented as a risk factor for unfavourable postoperative outcomes and increased perioperative complications.^21-24^ However, the impact of preoperative anaemia on inflammatory markers and their diagnostic utility in PJI remains underexplored. Recent advances in PJI pathophysiology and new diagnostic tools highlight its complexity. Chronic PJI is linked to increased local bone turnover, complicating diagnosis.^34^ Anaemia-induced alterations in baseline inflammatory markers may compromise diagnostic accuracy, potentially yielding both false-positive and false-negative results. Recent studies have highlighted the diagnostic potential of coagulation markers, including D-dimer, fibrinogen, and FDP in PJI;^15,35,36^ however, their diagnostic thresholds remain undefined in anaemic populations. Additionally, Macheras et al^37^ have demonstrated the potential utility of intraoperative calprotectin lateral flow immunoassay in guiding surgical decision-making between one- and two-stage revision arthroplasty in patients with suspected PJI.

Synovial fluid analysis is the diagnostic gold standard for PJI, with high sensitivity and specificity. However, limited evidence exists on the impact of anaemia on synovial biomarkers.^38,39^ This knowledge gap emphasizes the need for a comprehensive evaluation of anaemia’s influence on both serum and synovial markers in PJI diagnosis. Our findings indicate that preoperative anaemia is associated with statistically significant increases in several inflammatory biomarkers, with a more pronounced effect compared with aseptic failure. Anaemic PJI patients had higher serum and synovial levels of CRP, ESR, D-dimer, IL-6, SF-WBC, and SF-PMN% compared to non-anaemic patients. In AF, anaemia only elevated ESR, D-dimer, and fibrinogen. This differential effect suggests that diagnostic thresholds for inflammatory markers may need adjustment in anaemic patients to optimize accuracy. Previous studies have shown that iron deficiency is independently associated with elevated inflammatory biomarkers, particularly CRP and IL-6.^26^ A systematic review revealed that anaemic patients with chronic conditions exhibited statistically significantly elevated inflammatory markers compared with non-anaemic patients,^40^ while Li et al^41^ identified that elevated IL-6 and CRP levels independently predicted higher odds of anaemia. Our microbiological analysis revealed that while Gram-positive organisms predominated similarly in both cohorts (62.6% vs 54.1%), anaemic patients showed a higher rate of MRSA infections (6.1% vs 0.8%) but fewer polymicrobial infections (22.1% vs 33.6%), suggesting potential implications for antimicrobial strategies.

Anaemia affects PJI inflammatory markers: CRP, ESR, and IL-6 show higher accuracy and sensitivity in anaemic patients, requiring elevated thresholds. D-dimer has moderate utility, and procalcitonin has limited value regardless of anaemia status. Synovial fluid markers (WBC, PMN%) are consistent regardless of anaemia. In addition, stratification of PJI patients by BMI showed no significant differences in serum inflammatory marker levels among normal-weight, overweight, and obese groups (Supplementary Table i), indicating that BMI-related differences exert little measurable impact on these systemic inflammatory biomarkers. Prior studies show that these markers’ utility varies with host factors.^42^ CRP and ESR exhibit higher false-negative rates in chronic infections and immunocompromised conditions, with varying diagnostic thresholds.^8^ However, our study represents the first systematic analysis of inflammatory marker performance in anaemic patients with PJI.

The potential mechanisms underlying the association between anaemia and elevated inflammatory markers remain incompletely understood.^43^ Anaemia of inflammation, a common complication in chronic diseases including infections, directly influences immune cell function and cytokine production.^44,45^ During inflammation, increased hepcidin levels lead to iron sequestration in macrophages, creating a feedback loop that amplifies the inflammatory response.^46^ This mechanism may explain the elevated CRP and ESR levels observed in our anaemic PJI cohort. The relationship between iron deficiency anaemia and coagulation markers has been previously investigated. Jimenez et al^47^ demonstrated that iron deficiency induced thrombocytosis and elevated coagulation markers in experimental models. In our study, D-dimer showed suboptimal diagnostic utility despite anaemia-adjusted thresholds. Conversely, synovial fluid markers (elevated thresholds) demonstrated superior performance in anaemic patients, with increased specificity. This may reflect local inflammatory changes due to disrupted iron homeostasis, although further investigation is needed.

This study has several limitations. First, as a single-centre retrospective study, it is subject to inherent selection bias. Second, the small sample size, especially for synovial fluid analyses, may limit result robustness. Third, we assessed only conventional synovial inflammatory markers, not emerging ones like synovial CRP, α-defensin, d-lactate, or calprotectin. Lastly, inflammatory marker performance was not stratified by anaemia type or severity. Future multicentre prospective studies should validate these anaemia-adjusted diagnostic thresholds and evaluate a broader range of synovial biomarkers. Additionally, investigating the impact of anaemia type and severity on biomarker performance could further refine diagnostic strategies for PJI.

## Data Availability

The data that support the findings for this study are available to other researchers from the corresponding author upon reasonable request.

## References

[1] Ma T Jiao J Guo D-W Lv S-Z Zhang D Hou D-C Incidence of periprosthetic joint infection after primary total knee arthroplasty shows significant variation: a synthesis of meta-analysis and bibliometric analysis J Orthop Surg Res 2024 19 1 649 10.1186/s13018-024-05099-8 39396015 PMC11470562

[2] Jin X Gallego Luxan B Hanly M et al. Estimating incidence rates of periprosthetic joint infection after hip and knee arthroplasty for osteoarthritis using linked registry and administrative health data Bone Joint J 2022 104-B 9 1060 1066 10.1302/0301-620X.104B9.BJJ-2022-0116.R1 36047015 PMC9948458

[3] Szymski D Walter N Hierl K Rupp M Alt V Direct hospital costs per case of periprosthetic hip and knee joint infections in Europe - a systematic review J Arthroplasty 2024 39 7 1876 1881 10.1016/j.arth.2024.01.032 38266688

[4] Premkumar A Kolin DA Farley KX et al. Projected economic burden of periprosthetic joint infection of the hip and knee in the United States J Arthroplasty 2021 36 5 1484 1489 10.1016/j.arth.2020.12.005 33422392

[5] Denyer S Eikani C Sheth M Schmitt D Brown N Diagnosing periprosthetic joint infection Bone Jt Open 2023 4 11 881 888 10.1302/2633-1462.411.BJO-2023-0094.R1 37984446 PMC10659814

[6] Saleh A George J Faour M Klika AK Higuera CA Serum biomarkers in periprosthetic joint infections Bone Joint Res 2018 7 1 85 93 10.1302/2046-3758.71.BJR-2017-0323 29363518 PMC5805828

[7] Akkaya M Akcaalan S Perrone FL Sandiford N Gehrke T Citak M Organism profile and C-reactive protein (CRP) response are different in periprosthetic joint infection in patients with hepatitis Arch Orthop Trauma Surg 2024 144 1 341 346 10.1007/s00402-023-05059-7 37742285

[8] Lazarides AL Vovos TJ Reddy GB et al. Traditional laboratory markers hold low diagnostic utility for immunosuppressed patients with periprosthetic joint infections J Arthroplasty 2019 34 7 1441 1445 10.1016/j.arth.2019.03.013 30930152

[9] Kheir MM Tan TL Shohat N Foltz C Parvizi J Routine diagnostic tests for periprosthetic joint infection demonstrate a high false-negative rate and are influenced by the infecting organism J Bone Joint Surg Am 2018 100-A 23 2057 2065 10.2106/JBJS.17.01429 30516629

[10] Luger M Böhler C Puchner SE et al. Serum albumin-to-globulin ratio and CRP-to-albumin ratio did not outperform serum CRP in diagnosing periprosthetic joint infections Bone Joint Res 2024 13 8 372 382 10.1302/2046-3758.138.BJR-2024-0032.R1 39084635 PMC11290946

[11] Parvizi J Tan TL Goswami K et al. The 2018 definition of periprosthetic hip and knee infection: an evidence-based and validated criteria J Arthroplasty 2018 33 5 1309 1314 10.1016/j.arth.2018.02.078 29551303

[12] Shahi A Kheir MM Tarabichi M Hosseinzadeh HRS Tan TL Parvizi J Serum D-dimer test is promising for the diagnosis of periprosthetic joint infection and timing of reimplantation J Bone Joint Surg Am 2017 99-A 17 1419 1427 10.2106/JBJS.16.01395 28872523

[13] Wang R Zhang H Ding P Jiao Q The accuracy of D-dimer in the diagnosis of periprosthetic infections: a systematic review and meta-analysis J Orthop Surg Res 2022 17 1 99 10.1186/s13018-022-03001-y 35172830 PMC8848660

[14] Tarabichi S Goh GS Baker CM Chisari E Shahi A Parvizi J Plasma D-dimer is noninferior to serum C-reactive protein in the diagnosis of periprosthetic joint infection J Bone Joint Surg Am 2023 105-A 7 501 508 10.2106/JBJS.22.00784 36758110

[15] Chen X Xie J Li Y Jian Z Li H Yan Q Limited value of coagulation parameters in diagnosing periprosthetic joint infection Int Orthop 2022 46 10 2189 2194 10.1007/s00264-022-05495-x 35790548

[16] Xu H Xie J Huang Q Lei Y Zhang S Pei F Plasma fibrin degradation product and D-dimer are of limited value for diagnosing periprosthetic joint infection J Arthroplasty 2019 34 10 2454 2460 10.1016/j.arth.2019.05.009 31155460

[17] Cai Y Liang J Chen X et al. Synovial fluid neutrophil extracellular traps could improve the diagnosis of periprosthetic joint infection Bone Joint Res 2023 12 2 113 120 10.1302/2046-3758.122.BJR-2022-0391.R1 36718647 PMC9950667

[18] Jandl NM Kleiss S Mussawy H Beil FT Hubert J Rolvien T Absolute synovial polymorphonuclear neutrophil cell count as a biomarker of periprosthetic joint infection Bone Joint J 2023 105-B 4 373 381 10.1302/0301-620X.105B4.BJJ-2022-0628.R1 36924172

[19] Bailey A Eisen I Palmer A et al. Preoperative anemia in primary arthroplasty patients-prevalence, influence on outcome, and the effect of treatment J Arthroplasty 2021 36 7 2281 2289 10.1016/j.arth.2021.01.018 33549420

[20] Bierbaum BE Callaghan JJ Galante JO Rubash HE Tooms RE Welch RB An analysis of blood management in patients having a total hip or knee arthroplasty J Bone Joint Surg Am 1999 81-A 1 2 10 10.2106/00004623-199901000-00002 9973048

[21] Zhang HC Zhang Y Dai HB Wu D Xu B Preoperative anemia and complications after total joint arthroplasty: a systematic review and meta-analysis Eur Rev Med Pharmacol Sci 2022 26 20 7420 7430 10.26355/eurrev_202210_30011 36314312

[22] Zhang F-Q Yang Y-Z Li P-F et al. Impact of preoperative anemia on patients undergoing total joint replacement of lower extremity: a systematic review and meta-analysis J Orthop Surg Res 2024 19 1 249 10.1186/s13018-024-04706-y 38637795 PMC11027536

[23] Schmidt-Braekling T Sabri E Kim PR et al. Prevalence of anemia and association with outcome in joint arthroplasty – is there a difference between primary and revision cases? Arch Orthop Trauma Surg 2024 144 5 2337 2346 10.1007/s00402-024-05247-z 38416136

[24] Gu A Chen AZ Selemon NA et al. Preoperative anemia independently predicts significantly increased odds of short-term complications following aseptic revision hip and knee arthroplasty J Arthroplasty 2021 36 5 1719 1728 10.1016/j.arth.2020.10.061 33248920

[25] Wieczorek M Schwarz F Sadlon A et al. Iron deficiency and biomarkers of inflammation: a 3-year prospective analysis of the DO-HEALTH trial Aging Clin Exp Res 2022 34 3 515 525 10.1007/s40520-021-01955-3 34533774 PMC8894209

[26] Lu M Sing DC Kuo AC Hansen EN Preoperative anemia independently predicts 30-day complications after aseptic and septic revision total joint arthroplasty J Arthroplasty 2017 32 9S S197 S201 10.1016/j.arth.2017.02.076 28390884

[27] Sukhonthamarn K Tan TL Xu C et al. Determining diagnostic thresholds for acute postoperative periprosthetic joint infection J Bone Joint Surg Am 2020 102-A 23 2043 2048 10.2106/JBJS.20.00257 32941311

[28] Talsma DT Ploegmakers JJW Jutte PC Kampinga G Wouthuyzen-Bakker M Time to positivity of acute and chronic periprosthetic joint infection cultures Diagn Microbiol Infect Dis 2021 99 1 115178 10.1016/j.diagmicrobio.2020.115178 33017799

[29] Robertson FM Clement ND Preoperative anemia is associated with worse joint-specific postoperative outcomes, but is not associated with health-related quality of life or patient satisfaction after total knee arthroplasty J Arthroplasty 2023 38 1 51 59 10.1016/j.arth.2022.07.010 35921998

[30] Fontalis A Yasen AT Giebaly DE Luo TD Magan A Haddad FS Optimizing debridement and implant retention in acute periprosthetic joint infections Bone Joint J 2024 106-B 12 1377 1384 10.1302/0301-620X.106B12.BJJ-2024-0282.R1 39615530

[31] Li Y Wuermanbieke S Wang F et al. Efficacy and safety of intra-articular-only meropenem after one-stage revision for treating Escherichia coli -induced periprosthetic joint infection in a rat model Bone Joint Res 2024 13 10 546 558 10.1302/2046-3758.1310.BJR-2024-0119.R1 39362652 PMC11449542

[32] Li Y Zhang X Ji B et al. One-stage revision using intra-articular carbapenem infusion effectively treats chronic periprosthetic joint infection caused by Gram-negative organisms Bone Joint J 2023 105-B 3 284 293 10.1302/0301-620X.105B3.BJJ-2022-0926.R1 36854321

[33] Straub J Staats K Vertesich K Kowalscheck L Windhager R Böhler C Two-stage revision for periprosthetic joint infection after hip and knee arthroplasty Bone Joint J 2024 106-B 4 372 379 10.1302/0301-620X.1064.BJJ-2023-0638.R2 38555938

[34] Hinz N Butscheidt S Jandl NM et al. Increased local bone turnover in patients with chronic periprosthetic joint infection Bone Joint Res 2023 12 10 644 653 10.1302/2046-3758.1210.BJR-2023-0071.R1 37813394 PMC10562080

[35] Xu H Zhou J Huang Q Huang Z Xie J Zhou Z Unreliability of serum- or plasma-based assays of D-dimer or fibrin (fibrinogen) degradation product for diagnosing periprosthetic joint infection: a prospective parallel study Orthop Surg 2024 16 1 29 37 10.1111/os.13935 37975182 PMC10782268

[36] Xu H Xie J Wang D Huang Q Huang Z Zhou Z Plasma levels of D-dimer and fibrin degradation product are unreliable for diagnosing periprosthetic joint infection in patients undergoing re-revision arthroplasty J Orthop Surg Res 2021 16 1 628 10.1186/s13018-021-02764-0 34666806 PMC8524877

[37] Macheras GA Argyrou C Tzefronis D et al. Intraoperative calprotectin lateral flow immunoassay can assist decision-making between one- and two-stage revision total hip arthroplasty for patients with suspected periprosthetic joint infection Bone Joint J 2024 106-B 5 Supple B 118 124 10.1302/0301-620X.106B5.BJJ-2023-0848.R1 38688513

[38] Christopher ZK Braathen D Blackburn BE et al. Analysis of synovial fluid aspirations in aseptic loosening and instability after total knee arthroplasty J Arthroplasty 2025 40 7 1875 1880 10.1016/j.arth.2024.12.017 39706353

[39] Yadav AK Murhekar S Cinar EN Analysis of serum and synovial inflammatory markers in periprosthetic joint infections: a narrative review Cureus 2024 16 11 e72821 10.7759/cureus.72821 39493345 PMC11528397

[40] Hoepers AT Menezes MM Fröde TS Systematic review of anaemia and inflammatory markers in chronic obstructive pulmonary disease Clin Exp Pharmacol Physiol 2015 42 3 231 239 10.1111/1440-1681.12357 25641228

[41] Li J Chen W Wen X Jin X Zhu P Jiang C Association between inflammatory markers and anemia in patients with diabetic foot ulcer Biomark Med 2024 18 23 1037 1047 10.1080/17520363.2024.2421159 39535133 PMC11633438

[42] Ali ET Jabbar AS Mohammed AN A comparative study of interleukin 6, inflammatory markers, ferritin, and hematological profile in rheumatoid arthritis patients with anemia of chronic disease and iron deficiency anemia Anemia 2019 2019 3457347 10.1155/2019/3457347 31057960 PMC6463678

[43] Johnson EE Wessling-Resnick M Iron metabolism and the innate immune response to infection Microbes Infect 2012 14 3 207 216 10.1016/j.micinf.2011.10.001 22033148 PMC3270215

[44] De Domenico I Zhang TY Koening CL et al. Hepcidin mediates transcriptional changes that modulate acute cytokine-induced inflammatory responses in mice J Clin Invest 2010 120 7 2395 2405 10.1172/JCI42011 20530874 PMC2898601

[45] Marques O Weiss G Muckenthaler MU The role of iron in chronic inflammatory diseases: from mechanisms to treatment options in anemia of inflammation Blood 2022 140 19 2011 2023 10.1182/blood.2021013472 35994752

[46] Krawiec P Mroczkowska-Juchkiewicz A Pac-Kożuchowska E Serum hepcidin in children with inflammatory bowel disease Inflamm Bowel Dis 2017 23 12 2165 2171 10.1097/MIB.0000000000001245 28945637

[47] Jimenez K Leitner F Leitner A et al. Iron deficiency-induced thrombocytosis increases thrombotic tendency in rats Haematologica 2021 106 3 782 794 10.3324/haematol.2019.245092 32079699 PMC7928018

